# Outcomes of myasthenia gravis patients admitted to the Intensive Care Unit: Experience from a tertiary care center in Saudi Arabia

**DOI:** 10.1371/journal.pone.0328648

**Published:** 2025-07-28

**Authors:** Ahmed Attar, Hatoon Alshaikh, Ghadi Alharbi, Tala Aletani, Sara Alghamdi, Sarah Alzahrani, Osama Khojah, Rahma Alqthmi, Abdulrahman Althobaiti, Thamer Alaifan

**Affiliations:** 1 Department of Neurology, Ministry of the National Guard-Health Affairs, Jeddah, Saudi Arabia; 2 College of Medicine, King Saud bin Abdulaziz University for Health Sciences, Jeddah, Saudi Arabia; 3 King Abdullah International Medical Research Center, Jeddah, Saudi Arabia; 4 Department of Medicine, McMaster University, Hamilton, Ontario, Canada; 5 Intensive Care Unit Department, Ministry of the National Guard-Health Affairs, Jeddah, Saudi Arabia; University of Miami Miller School of Medicine: University of Miami School of Medicine, UNITED STATES OF AMERICA

## Abstract

**Background:**

Myasthenia gravis is an autoimmune disorder that affects the neuromuscular junction resulting in muscle weakness and fatigue. The aim of this study was to investigate the indications for and frequency of intensive care unit admissions in people with myasthenia gravis in addition to exploring the clinical presentations and outcomes during their admission.

**Methods and Findings:**

This retrospective study included all adult patients diagnosed with myasthenia gravis who were admitted to the intensive care unit in a six year period. Twenty-four patients with a diagnosis of myasthenia gravis were included in this study, with a total of 60 admissions. The median age at their first admission was 45 years with female predominance. Majority of the patients (87.5%) were acetylcholine antibody positive and 66.7% of patients had thymectomy. Myasthenic crisis was the most common reason for intensive care unit admission (63.3%). 80.5% of patients admitted with myasthenic crisis were treated with plasma exchange. During their admission, 45% of patients required mechanical ventilation. Complications during hospitalization were reported in 18.3% of patients, with mortality rate of 5% of all admissions.

**Conslusion:**

People with myasthenia gravis are commonly admitted to the intensive care unit due to myasthenic crises, however, there were minimal complications reported and a low mortality rate.

## 1. Background

Myasthenia gravis (MG) is an autoimmune neuromuscular disorder characterized by widespread or localized muscle weakness and fatigue [1]. MG is a disorder of the neuromuscular junction with incidence rates varying from 0.3 to 2.8 per 100,000 globally, with a median prevalence of about 10 per 100,000 individuals [2]. The disorder arises from antibodies targeting neuromuscular junction components, notably the anti-acetylcholine receptor (AChR) antibody, which reduces the number of available acetylcholine receptors. Another significant but less frequently found antibody is muscle-specific tyrosine kinase (MuSK) [1]. MG often starts with ocular symptoms, progressing to generalized weakness, including bulbar, respiratory, and limb muscles [3].

Myasthenic crisis, categorized as class V by the Myasthenia Gravis Foundation of America (MGFA), represents one of the most serious complications associated with MG. This condition is characterized by acute respiratory failure triggered by an exacerbation of MG symptoms, necessitating the use of mechanical ventilation (MV), and typically arises from weakness in the respiratory muscles but may also result from bulbar weakness leading to upper airway collapse [1,2,4]. Approximately 15% to 20% of individuals diagnosed with MG are likely to face a myasthenic crisis within the first few years of their diagnosis [2,5,6]. A retrospective analysis of 245 patients from Australia and New Zealand, who were admitted to the intensive care unit (ICU) with a primary diagnosis of MG, revealed that 37.1% of these patients needed MV, and the hospital mortality rate stood at 5.3% [7]. Additionally, a study in Saudi Arabia on 147 patients between 2010 and 2020 showed that 36.1% of people with MG (pwMG) had at least one exacerbation during their follow-up and were admitted to the ICU, corroborating previous findings in the region [8]. However, it is important to mention that pwMG are admitted to the ICU not only because of MG crises but also due to a wide array of medical and surgical problems which may or may not be associated with a crisis.

Generally, the effectiveness of treatment and the prognosis for individuals with MG differ based on various factors, including the patient’s clinical profile, the type of autoantibodies present, and the existence of any additional health conditions [9]. Additionally, the demographic profile of MG patients appears to be shifting, with an increasing incidence observed across older age groups in both genders, moving away from the previous commonality in younger women [10].

Currently, there is a lack of comprehensive data regarding pwMG, particularly those requiring admission to the ICU, in Saudi Arabia [11,12]. Accordingly, this study focuses on exploring the clinical presentations and outcomes of MG patients admitted to the ICU. It aims to estimate the rate of ICU admissions among MG patients, assess mortality, physical disability, and ventilation needs upon discharge, and identify the predominant reasons for their ICU admissions.

## 2. Methods

This retrospective cohort study employed a consecutive sampling technique to include all adult patients diagnosed with myasthenia gravis who were admitted to the ICU from January 2016 to January 2023 at King Abdulaziz Medical City in Jeddah, Saudi Arabia.

The ICD-10 code G70.0 “Myasthenia gravis” was used to retrieve and review MG cases. The records of patients who were admitted to the ICU department underwent an in-depth review and data collection. Patients who had been transferred from another hospital (ICU to ICU) or had congenital myasthenic syndrome were excluded from the study. At our institution, the diagnosis of MG was established through a combination of clinical features, serology testing (AChR-Ab or MuSK-Ab), and electrodiagnostic studies (repetitive nerve stimulation). Once diagnosed, pwMG were prescribed an acetylcholinesterase inhibitor and were followed up to assess their response. Those with an unsatisfactory response were then started on an immunosuppressive medication. All patients are assessed for thymus pathology. For MG crises, our institution is capable of providing intensive care and administer therapies such as plasmapheresis (PLEX), intravenous immunoglobulin (IVIg), and/or intravenous methylprednisolone (IVMP) as required. Pyridostigmine is held in pwMG during a suspected MG crisis. During hospitalization and subsequent outpatient follow-up, pwMG were evaluated for the need for maintenance immunosuppressive therapy. For those requiring maintenance immunosuppression, standard dosing regimens for rituximab, mycophenolate mofetil, and azathioprine were used. Rituximab was administered as two 1-gram intravenous infusions given two weeks apart, followed by 1-gram infusions every six months. The target dose for mycophenolate mofetil was 1,000–1,500 mg twice daily, while azathioprine was dosed at 2–3 mg/kg/day. PwMG are followed on an outpatient basis every six to twelve months, although more frequent follow-up may be required depending on the complexity of care and clinical status. In our institution, PwMG undergoing thymectomy receive IVIg 2 g/kg, administered over a period of two to five days prior to the procedure. The data was then collected using a structured data collection sheet that included demographic and clinical characteristics (age, gender, duration of the disease), serology tests (AchR and MuSK antibodies), thymus disease and management (chest computed tomography and pathology results, thymectomy, and thymectomy approach), frequency of emergency department visits and ICU admissions, reason for admission, treatments the patient were receiving at the time of ICU admission (e.g., pyridostigmine, prednisone, or steroid-sparing immunosuppressants), length of stay, and outcomes (mortality, dependency, and disposition). Dependency included pwMG who had respiratory (e.g., supplementary oxygen source) or functional (e.g., walker or wheelchair) dependency upon discharge from the ICU. For patients who were admitted to the ICU due to a myasthenic crisis, symptoms, etiologies, and treatment regimens were collected. Myasthenic crisis was defined as respiratory failure requiring MV or non-invasive ventilation (NIV) [6]. Regarding the decision to initiate NIV or MV in our institution, NIV was typically considered for patients with mild to moderate respiratory distress who retained adequate airway protection and had no significant bulbar dysfunction. In contrast, MV was initiated in cases of severe respiratory failure, inability to clear secretions, significant bulbar involvement, or failure to improve with NIV.

Statistical analysis was performed using JMP Statistical Software version 15.2.0 (SAS Institute, Cary, NC; a subsidiary of the SAS Institute). Frequencies and percentages were used to represent all categorical variables such as gender and clinical manifestations of myasthenic crises. Numerical variables such as age, number of ICU admissions, and the duration of ICU stay were represented using medians and interquartile ranges due to the non-parametric distribution of the data. Data was accessed by researches between Febuary, 2, 2024 and April 20, 2024. During data collection, authors had access to patient’s electronic medical records with include identifiers. All data are fully available without restriction in Supporting Information. Ethical approval was obtained from King Abdullah International Medical Research Centre’s Institutional Review Board (reference number: JED-23-427780-131608) on September 03, 2023, and the confidentiality of all patients’ data was ensured per the Declaration of Helsinki.

## 3. Results

Among the 52 pwMG following at our institution during the study period, 24 (46.6%) were admitted to the ICU, resulting in a total of 60 ICU admissions. These included 15 elective admissions, of which 53.3% were primarily for post-thymectomy observation, and 45 urgent admissions. The mean age of patients admitted to ICU was 52.3 ± 16.9 years, with a median age at first ICU admission of 45 years (range 26–86 years), and a female-to-male ratio of 3:1. Patients were admitted to the ICU a median of 2 times (range 1–10), with a median duration of 3 years from MG onset to the first ICU admission (range 1 day–28 years). Three patients (5%), all over the age of 60 years, required ICU management during their initial presentation with MG. The median age of MG diagnosis was 39 years (range 22–86 years). Late-onset MG was diagnosed in 25% of patients but represented 43.3% of ICU admissions. AChR antibodies and MuSK antibodies were detected in 87.5% and 16.8%, respectively. Notably, one patient tested positive for both AChR and MuSK antibodies. MuSK-MG represented 20% of ICU admissions. Thymus pathologies were identified in 62.5%, with thymoma being the most documented thymus pathology (41.8%), and history of thymectomy was reported in 66.7% of patients. The demographics, clinical characteristics, and emergency room visits due to myasthenic crisis are shown in Table 1.

**Table 1:** Demographics and clinical characteristics of patients with myasthenia gravis.

Variable	Median (IR) or frequency (%)
**Total number of patients**	24
**Total number of ICU admissions**	60
**Age at MG diagnosis in years**	39 (20.75)
**Age at first ICU admission in years**	47 (22.5)
**Sex: Female**	18 (75%)
**Antibody status:**	
Anti-AChR positive	20 (83.33%)
Anti-MuSK positive	3 (12.5%)
Double positive	1 (4.17%)
**Thymus pathology:**	
Thymoma	10 (41.67%)
Hyperplasia	4 (16.67%)
Atrophy	1 (4.17%)
None	10 (41.67%)
**Thymectomy (n = 16):**	
Videoscopic Thymectomy	6 (37.5%)
Total median sternotomy	3 (18.75%)
Unknown	7 (43.75%)
**ED visits due to myasthenic crisis**	1 (2.5)

Abbreviation: ICU: intensive care unit; AChR: acetylcholine receptor; MuSK: muscle-specific tyrosine-kinase; ED: emergency department.

Out of 60 ICU admissions, the initial reason for 63.3% of the ICU admissions was a myasthenic crisis. However, 3 out of the 8 patients who were admitted to ICU for post-thymectomy monitoring developed a myasthenic crisis during their ICU stay, making the total cases of myasthenic crises managed in the ICU 41 (68.3%). The most common triggers included infections (34.2%) and non-adherence to medications (7.3%). Majority of patients who developed myasthenic crises presented with both bulbar and limb symptoms (68.3%) upon their ICU admission. Of all ICU admissions, 61.7% pwMG were on one or more immunosuppressive medication prior to their admission. However, almost all of the patients who developed an exacerbation were either on acetylcholinesterase inhibitors (97.6%) or immunosuppressives (90.2%) before the exacerbation. PLEX, typically over five session, was the predominant treatment in myasthenic crises, used in 80.5% of these cases. For respiratory support, 27 (45%) patients required NIV, and 18 (30%) required MV. Of patients who needed respiratory support during their ICU admission, 66.7% had a myasthenic crisis. Eleven patients (18.3%) experienced complications during hospitalization, including pneumonia (6.7%), sepsis (5%), and pleural effusion (1.7%). The mortality rate was 5% of ICU admissions, all of whom were over the age of 60 and were admitted to ICU due to myasthenia exacerbations. Causes of mortality included multiorgan failure due to sepsis (66.7%) and multiorgan failure of other origin (33.3%). Table 2. shows the reasons for ICU admissions, main symptoms of myasthenic crisis upon ICU admission, as well as the management and outcomes of pwMG in the ICU.

**Table 2:** Management of patients with myasthenia gravis in ICU.

Variable	Frequency (%) (n = 60)	
**Age: mean ± SD**	52.3 ± 16.86	
**ICU stay (days): Median (IR)**	4 (4.75)	
**Initial reason for admission:**		
Myasthenic crisis	38 (63.33%)*	
Post-thymectomy monitoring	8 (13.33%)	
Post-operative monitoring	5 (8.33%)	
Infections	4 (6.66%)	
Respiratory failure	3 (5%)	
Medical Procedure	2 (3.33%)	
**Nasogastric tube**	16 (26.67%)	
**NIV**	27 (45%)	
**Duration of NIV in days: Median (IR)**	5 (10)	
**MV**	18 (30%)	
**Duration of MV in days: Median (IR)**	2 (4)	
**Main symptoms of myasthenic crisis upon ICU admission (n = 41)**	**Bulbar and limbs:**	28 (68.29%)
	Dysphagia	18 (43.90%)
	Weak Jaw/ Facial muscles	13 (31.71%)
	Dysphonia	8 (19.51%)
	Slurred Speech	7 (17.07%)
	Difficulty handling secretions	3 (7.32%)
	Nasal regurgitation	2 (4.88%)
	Aspiration of secretions	2 (4.88%)
	**Limbs**	13 (31.71%)
**Treatment before myasthenic crisis (n = 41):**	**Acetylcholinesterase inhibitors (pyridostigmine)**	38 (97.56%)
	**Immunosuppressives (n = 37)**	
	Prednisone	30 (81.08%)
	Azathioprine	9 (24.32%)
	Mycophenolate Mofetil	9 (24.32%)
	Rituximab	8 (21.62%)
**Myasthenic crisis therapy (n = 41):**		
PLEX alone	29 (70.73%)	
IVIg alone	7 (17.07%)	
PLEX + IVIg	2 (4.87%)	
PLEX + IVIg + IVMP	1 (2.43%)	
PLEX + IVMP	1 (2.43%)	
IVIg + IVMP	1 (2.43%)	
**Dependency**	28 (47.45%)	
**ICU mortality**	3 (5%)	

*Three patients admitted for post-thymectomy monitoring went into a myasthenic crisis.

Abbreviation: ICU: intensive care unit; Bi-PAP: bilevel positive airway pressure; NIV: non-invasive ventilation; MV: mechanical ventilation; MG: myasthenia gravis; PLEX: plasma exchange; IVIg: intravenous immunoglobulin; IVMP: intravenous methylprednisolone.

## 4. Discussion

This study describes the clinical course and ICU management of pwMG, highlighting the elevated female to male ratio 3:1, high admission rate of pwMG to the ICU (46.2%), predominance of myasthenic crises as cause for ICU admissions (63.3%), utilization of MV (30%), and notable mortality rate (5%).

Epidemiological studies have shown that MG has a bimodal distribution, typically affecting young females and older males [13]. The average age of pwMG who require ICU management generally range between 50 and 60 years which was reported in different studies from different regions, including Serbia, the United States, Australia, New Zealand, the United Kingdom, and in our study [2,5,7,13,14]. Our findings also showed a female predominance similar to what was found by previous studies [2,5,7,14,15]. While it could be hypothesized that females with MG in their 50s and 60s have a more severe disease, a multicenter retrospective study conducted by Neumann et al. in Germany, which included 250 pwMG who required MV, instead found a male predominance [16]. These epidemiological features of MG remain puzzling and require large studies that take into account ethnic, social, and economical factors into consideration.

While there were many similarities when we compared pwMG admitted to the ICU with the general MG population in Saudi Arabia, there were also some differences. Algahtani et al. and Ali et al described the characteristics of MG in tertiary care centers in Saudi Arabia and found an ICU admission rate of 20.1% and 13.6%, respectively [8,12]. The notable discrepancy between these rates and those in our study may be attributed to a tendency in previous studies to report only ICU admissions resulting from MG crises, whereas our study also includes elective admissions. The mean age of pwMG in Saudi Arabia is around 33.5 years to 34.2 years, which is lower than those who require ICU management (50–60) [8,12]. This age-related difference highlights the importance of considering age when assessing pwMG who could be admitted to the ICU.

Akin to the literature, our study showed that AChR-MG was the predominant type among patients admitted to the ICU, representing approximately 80% of patients followed by MuSK-MG [2]. While in the literature, seronegative MG was reported to represent 6.9% of pwMG admitted to the ICU, none of the patients reported our study had a seronegative disease [2]. This discrepancy likely results from the small sample size of our study. The antibody status does not seem to affect the outcome of NIV trial, duration of ventilation, or survival in patients with myasthenic crisis, but there are clinical variations among patients with different antibodies [16,17]. Seronegative MG is thought to be milder than the seropositive counterpart, and MuSK-MG is the more severe type. MuSK-MG typically accounts for 5–8% of MG cases and approximately 10% pwMG admitted to the ICU [2,18]. In our study, MuSK-MG represented 12.5% (without accounting double positive) of pwMG admitted to the ICU and MuSK-MG represented 20% of ICU admissions. This increase is likely because MuSK-MG is associated with an increased risk of myasthenic crises [18].

One third of ICU admissions in our study were associated with an underlying infection, all of whom were using an immunosuppressive agent. Infections continue to be a leading factor in emergency department visits, hospitalizations, and ICU admission for pwMG [2,5,14,19,20]. A significant proportion of these infections, which contribute to 15% of myasthenic crises, are preventable through vaccination [20,21]. This link between infections and myasthenic crises is likely due to the interplay between immune dysfunction in pwMG, use of immunosuppressive medications, and complications associated with muscle weakness [21]. Immunosuppression is a cornerstone of MG treatment; however, it significantly increases the risk of infections, which can in turn lead to crises [22]. Currently, there are no specific guidelines regarding vaccinations or the use of prophylactic antibiotics in pwMG, highlighting the need for further research in this area. It is essential to educate pwMG about the importance of vaccines, infection prevention practices such as hand hygiene, and muscle weakness rehabilitation.

In our patients, crises were mainly managed with PLEX (80.5%), supplemented, in select cases, by IVIg or IVMP. This selection of treatment modalities reflected a personalized approach based on factors, such as prior treatment responses and individual patient factors (e.g., PLEX is not suitable for patients with sepsis and IVIg cannot be used in those with renal failure) [23]. Additionally, experts generally suggest that PLEX has a quicker action and might be more effective than IVIg in cases of impending or manifest myasthenic crisis [23]. There is growing emphasis on preventing myasthenic crises rather than solely managing them in the ICU. Immunosuppressive therapy constitutes the cornerstone of long-term myasthenia gravis management, with corticosteroids serving as a primary agent for inducing clinical remission [24]. Following stabilization, gradual tapering of corticosteroids is pursued to mitigate adverse effects. To facilitate steroid-sparing strategies and minimize cumulative corticosteroid exposure, adjunctive immunosuppressive agents such as azathioprine, rituximab, mycophenolate mofetil, and tacrolimus are frequently employed [24]. In our cohort, most of the patients who were admitted to the ICU due to an exacerbation were on steroids and/or steroid-sparing agent. However, time of initiation of immunospuression was not collected. Neverthless, early consideration of interventions such as thymectomy, immunosuppression for ocular MG, and Rituximab for MuSK-MG is recommended [25].

Non-invasive ventilation was used in 45% of patients in this study. NIV can be beneficial for patients with less severe symptoms of MG, who are requiring respiratory support, by preventing the need for intubation and reducing complications, such as atelectasis [26]. Given the pathophysiology of ventilatory failure in MG, early initiation of NIV at the onset of inspiratory muscle fatigue—before diaphragmatic weakness and overt hypoventilation occur—may prevent progression to severe respiratory failure and avoid intubation [16]. NIV has been effective in preventing intubation in select patients with myasthenic crisis, with a success rate of 38% [15]. It has been suggested that in suitable patients, NIV can offer adequate respiratory support until acute treatments such as plasma exchange take effect [16,27].

In our study, 33% of patients who were on NIV did not require invasive MV. According to a retrospective analysis of pwMG and respiratory failure, the use of NIV was associated with lower rates of pulmonary complications and length of ICU stay [26]. Furthermore, the number of patients that required invasive MV during their admission was notably lower than reported figures in the literature [2,7,15], which may be due to variability in patients’ response to NIV or extent of respiratory compromise. These findings underscore the substantial respiratory challenges inherent to MG, emphasizing the imperative for vigilant monitoring and timely interventions to mitigate the risk of respiratory morbidity and optimize patient outcome. During the patients’ stay in the ICU, 18.3% experienced complications such as pneumonia, sepsis, or other etiologies, with pneumonia being the most reported complication; similar to prior investigations [2,16]. Patients with severe MG exacerbations who do not require mechanical ventilation have a better outcome. However, the association between MV and mortality among pwMG remains uncertain. In a retrospective study conducted by Al-bassam et al. in Australia and New Zealand, which included 245 pwMG admitted to the ICU, no significant association between MV and mortality was observed (*P* = 0.2) [7].

In addition, effective management of MG crises requires careful consideration of comorbidities among pwMG, as previous research has shown a link between a high number of comorbidities and prolonged MV [2,16]; indicating that a decision to start MV should not be rushed in patients with multiple comorbidities.

The ICU mortality rate related to MG ranged in the literature from 2.4% to 18.6% but could be as high as 27.3% in limited resource setting [7,13,28,29]. Epidemiological studies estimated in-hospital mortality between 2.2% and 4.7%, with overall disease-specific mortality ranging from 5% to 9% [3,5]. In our study, the ICU mortality rate was 5% all of whom were admitted to ICU due to myasthenia exacerbation. This notable disparity in mortality rates between institutes with limited resources and others highlights the critical impact of healthcare infrastructure and resource allocation on patient outcomes across different regions. Furthermore, all patients who died during their ICU stay in this study were over the age of 60. This aligns with previous research, which has consistently reported advanced age as an independent predictor of mortality [5,28,30]. Additionally, in accordance with earlier studies, the cause of death in the three patients in this study was related to sepsis or other underlying factors which led to multiorgan failure [5,31]. Existing literature delves into various factors that could potentially contribute to the development of multiorgan failure in individuals with MG; the chronic nature of MG and its adverse effects on respiratory function could culminate in respiratory compromise, which may not only exacerbate sepsis, but also play a contributory role in the onset of multiorgan dysfunction [31,32]. In this study, all three patients who died during ICU admission had received PLEX therapy. Two of these deaths were attributed to multi-organ failure secondary to sepsis, and one to other causes. Given the retrospective nature of the study and the lack of information regarding the timing of PLEX administration in relation to onset or resolution of infection, no conclusions can be drawn about the role of PLEX in these settings. Among the 14 patients who experienced infection-related myasthenic exacerbation, 11 received PLEX, but it is unclear whether treatment was initiated before or after the resolution of infection. Without these details, it is difficult to determine whether PLEX contributed to clinical improvement.

### 4.1. Limitations

Several limitations warrant cautious interpretation of our findings. Firstly, the relatively small sample size, compounded by the retrospective nature of the study, and the study’s reliance on existing medical records may limit the generalizability of findings and introduce variability in data quality. This constrained sample size also precluded the performance of inferential analysis, necessitating reliance solely on descriptive analysis. Additionally, the lack of differentiation between early-onset and late-onset MG subtypes in our analysis may affect the generalizability of findings related to disease onset and treatment responses. The ICU admission rate reported in our study may reflect a sampling bias inherent to our institution’s role as a tertiary care center and specialized neuroscience referral hub. We receive referrals from across the Western and Southern regions of Saudi Arabia, with many cases representing refractory disease, requiring advanced or specialized neuro-interventions, or necessitating direct ICU-to-ICU transfer. As such, our patient population is inherently skewed toward more severe and complex presentations, which may overestimate ICU admission rates compared to the general population of patients with similar conditions.. Finally, absence of a control group limits the ability to establish causal relationships between variables.

## 5. Conclusion

This study highlights the characteristics of a vulnerable group who is susceptible to ICU admissions and associated complications. Approximately half of pwMG following at our institution required one or more ICU admission, with a female predominance. Myasthenic crisis was the most common cause for ICU admission in this study, with a third having an underlying infection as the trigger. It is essential to educate pwMG about the importance of vaccines and infection prevention practices and further research is needed on prophylactic measures for common infections affecting pwMG. Furthermore, the majority of patients admitted were on ventilator support, however only one third required invasive mechanical ventilation.

## Supporting information

S1 Data De-identified raw data of pwMG admitted to the Intensive Care Unit. (XLSX)

## Abbreviations

MG: Myasthenia gravis
AChR: Acetylcholine receptor
MuSK: Muscle-specific tyrosine kinase
MGFA: Myasthenia gravis foundation of America
MV: Mechanical ventilation
ICU: intensive care unit
PwMG: People with myasthenia gravis
PLEX: Plasmapheresis
IVIg: Intravenous immunoglobulin
IVMP: Intravenous methylprednisolone
NIV: non-invasive ventilation
